# *Ex Vivo* Cytokine Release and Pattern Recognition Receptor Expression of Subjects Exposed to Dampness: Pilot Study to Assess the Outcome of Mould Exposure to the Innate Immune System

**DOI:** 10.1371/journal.pone.0082734

**Published:** 2013-12-10

**Authors:** Stefanie Punsmann, Verena Liebers, Anne Lotz, Thomas Brüning, Monika Raulf

**Affiliations:** Institute of Prevention and Occupational Medicine, German Social Accident Insurance, Institute of the Ruhr-Universität Bochum, Bochum, Germany; Leiden University Medical Center, Netherlands

## Abstract

In rooms with moisture damage, the indoor air can be enriched with microorganisms causing a variety of symptoms. Due to the highly diverse composition of bioaerosols and the multiple effects on humans, an assessment of the health risk is not sufficiently possible. The aim of this study was to characterize the features of innate immunity using blood from subjects exposed to moisture damage compared to control subjects living in houses without visible moisture damage. We investigated the expression of TLR-2, TLR-4 and dectin-1 on the surface of monocytes from both fresh blood and after *in vitro* stimulation with the model substances *E. coli* endotoxin, zymosan A, Pam_3_Cys and *Aspergillus versicolor* in 25 exposed subjects and 25 control subjects. *In vitro* stimulation of whole blood with the same components was performed for 20 h and the release of inflammatory mediators IL-8 and IL-1β were quantified. In addition to an enhanced number of blood leucocytes, the expression of the receptors TLR-2, TLR-4 and dectin-1 on blood monocytes was significantly enhanced in exposed subjects. In contrast, no different alteration in expression was detected between exposed and control group after *in vitro* stimulation with the model substances. The release of IL-8 and IL-1β after stimulation of whole blood with *A. versicolor* was increased in subjects exposed to moisture damage. Furthermore, in the exposed subjects the IL-1β release was significantly enhanced after *in vitro* stimulation with *E. coli* endotoxin (1000 pg/mL). In conclusion, features of the innate immune system (receptor expression and mediator release of monocytes) are altered in subjects exposed to moisture damage which may be a potential explanation for the increased incidence of respiratory health diseases observed in these populations.

## Introduction

Knowledge on the health impact of bioaerosols caused by indoor exposure to dampness is limited. However, a recent comprehensive review by Tischer, Chen, and Heinrich 2011 summarized the available evidence, which suggests that home environment with visible mould and mould-spore exposure increase the risk of allergic respiratory health problems in children. The authors recommended that future research should focus on the effects of exposure to mould-derived components and specific microbial markers in the home, in combination with new assessment techniques including molecular methods [1]. Concentration of moulds, such as *Aspergillus versicolor*, *Penicillium chrysogenum* and *Cladosporium sphaerospermum*, and microbial components such as LPS and β-(1,3)-glucan were elevated in rooms with moisture damage [2,3]. The continued exposure to these bioaerosols may increase the risk of developing asthma, bronchitis, rhinosinusitis or hypersensitivity pneumonitis [4]. 

The complex composition of bioaerosols in damp houses with visible mould and mould spores is difficult to assess. In some recent studies, measurement of mould components, such as (1,3)-ß-D-glucan and extracellular polysaccharides (EPS) in house dust samples were used as surrogates for mould exposure [5,6]. Methods which detect the health-related properties in their entirety are still required. One possibility is the use of the whole blood assay - an *in vitro* system that measures both the pyrogenic and pro-inflammatory properties of complex samples, for example, those collected after indoor exposure to dampness [7]. 

In addition to bacterial components, both fungi and fungal components can stimulate the production of pro-inflammatory cytokines in human whole blood [8]. The cytokine release in fresh blood is more dependent on inter- than on intra-individual differences [9]. Various characteristics of the blood donor, such as age or gender, may be responsible for the variability [10]. Moreover, it is also possible that this variability is influenced by the individual biological susceptibility of the responder induced by the test stimulus. For example, high occupational exposure to endotoxin is reflected by high *ex vivo* cytokine release that is detected after *in vitro* stimulation of whole blood [11].

The knowledge on how exposure to bioaerosols in rooms with moisture damage is linked to the biological susceptibility of inhabitants exposed to the components of these bioaerosols is currently limited.

Many bioaerosol components have pathogen-associated molecular patterns (PAMP) that are recognized by pattern recognition receptors (PRRs). One of these ingredients is endotoxin, a component of the cell wall of Gram-negative bacteria. Due to the occurrence of endotoxin in bioaerosols, exposure via inhalation can occur at home, at the workplace, and even outdoors [12–14]. For example, elevated endotoxin concentrations have been reported in rooms after moisture damage [3]. 

Members of the Toll-like receptor (TLR) family are involved in response to bioaerosol components. TLR-4 plays a crucial role in the recognition and activation of cells after endotoxin exposure [15]. Typical TLR-2 ligands include lipoproteins from Gram-positive bacteria, which are often detected in bioaerosols from rooms with moisture damage [16]. The synthetic TLR-2/TLR-1 ligand, lipopeptid(*S*)-(2,3-bis(palmitoyloxy)-(2*RS*)-propyl)-*N*-palmitoyl-(R)-Cys-(*S*)-Ser(*S*)-Lys_4_-OH Trihydrocloride (Pam_3_Cys), is often used as a model for TLR-2 ligands. TLR expression on the cell surface is a dynamic process that is dependent on various factors, such as pathogen exposure, cytokines micro-environment, and other environmental conditions [17].

In addition to endotoxin and Gram-positive bacteria, β-1,3-glucan is also found in bioaerosols, with especially elevated concentrations in bioaerosols from rooms with moisture damage [3]. Zymosan A is a cell wall component of the yeast species *Saccharomyces cerevisiae*, and the main component of Zymosan A is β-1,3-glucan supplemented by minor components such as mannan and chitin [18]. Zymosan A can act as a ligand for dectin-1 or the receptor complex of TLR-2/TLR-6, which upon binding induces various immune reactions [19]. Dectin-1 is described as the primary receptor for both soluble, as well as particulate β-glucans from fungi, plants and bacteria [20]. The expression level of dectin-1 on the cell surface is influenced by cellular maturation, cytokines, steroids and the concentration of β-glucans [21]. One of the most commonly found moulds in rooms with moisture damage is *Aspergillus versicolor* [2,22]. Although not every exposure to this fungus leads to health problems, a variety of diseases such as rhinosinusitis, aspergillosis or asthma can be triggered [23]. 

Therefore, the aim of this study was to estimate the differences between cellular cytokine release and PRR expression in subjects living in homes with moisture damage and suffering from health effects compared to subjects who live without visible moisture damage. To characterize the effects on the human immune system, the individual mediator release (IL-β, TNF-α and IL-8) in fresh whole blood after *in vitro* stimulation with fungal and bacterial model substances (endotoxin, Zymosan A, Pam_3_Cys, *A. versicolor*) was studied in moisture damage-exposed and non-exposed subjects. Using flow cytometry, the individual receptor expression, both in non-stimulated whole blood cells and after stimulation with the model compounds was investigated. In addition, serum parameters such as interleukin (IL)-6, IL-8 and CC16 (clara cell protein), and plasma parameters such as the bactericidal permeability-increasing protein (BPI), lipopolysaccharide-binding protein (LBP), and sCD14 were also studied in both groups.

##  Materials and Methods

### Subjects

In total, fifty subjects were included in the study - 25 subjects exposed to moisture damage and 25 subjects without visible mould exposure in their homes (control group). All subjects completed a questionnaire concerning mould exposure in their dwellings together with existing health effects (rhinitis, eye problems, asthmatic symptoms, and headache). Based on this questionnaire, the classification as exposed subjects and controls was conducted. Subjects were classified as exposed when visible mould in their dwellings was greater than 210 x 297 mm (DIN A4). Subjects’ characteristics like age, gender, smoking and atopy are shown in Table 1. The subjects have no familial relation to each other. The study was reviewed and approved by the ethics committee at the Ruhr-University Bochum, and all subjects gave their written consent to participate.

**Table 1:** Characteristics of the study group.

**Category**	**Exposed**	**Controls**
Number of subjects: n	25	25
Age (yrs): mean ± SD (range)	35 ± 12 (20 - 63)	30 ± 9 (21 - 53)
Male subjects: n (%)	9 (36%)	11 (44%)
Non-Smokers: n (%)	21 (84%)	22 (88%)
Smokers: n (%)	4 (16%)	3 (12%)
Atopic: n (%) (Specific IgE sx1 ≥ 0,35 kU/L)	10 (40%)	10 (40%)
Total IgE [kU/L]: median (interquartile range)	31.9 (12.6-140.5)	43.8 (18.7-108.5)
Total IgE ≥ 100 kU/L: n (%)	8 (32%)	7 (28%)
Specific IgE mx1 ≥ 0.35 kUA/L: n (%)	2 (8%)	1 (4%)
Specific IgG Gmx6 ≥ 30 mg/L: n (%)	0 (0%)	0 (0%)

Study group consisted of subjects exposed to moisture damage and subjects not exposed to moisture damage (controls).

### Exposure assessment

Passive airborne dust was sampled with an electrostatic dust fall collector (EDC) for 14 days in the room with the moisture damage [24]. 18 of the 25 subjects were poised to lay out one EDC in the room with the moisture damage. Extracts of the dusts collected with EDC were prepared by shaking filters for 1 h in 20 mL PBS with 0.05% Tween, followed by collection of the fluid, and centrifugation for 15 min at 3000 g. The resulting supernatant was used for *A. versicolor*, *Penicillium chrysogenum* and β-(1,3)-Glukan quantification as described previously [25–27]. In addition, for endotoxin determination extracts without Tween were prepared. The determination was performed in the supernatants with Endochrome K (Charles River, Wilmington, USA) according to the manufacturer’s recommendations. Results in EU/mL were converted to square meters, calculating the dilution factor (20 mL) and the exposed area (0.0209 m^2^) of the tissue.

### Stimuli and reagents

*E. coli* O113:H10 control standard endotoxin (Cape Cod, East Fastmouth, USA), (S)-(2,3-bis(palmitoyloxy)-(2RS)-propyl)-N-palmitoyl-(R)-Cys-(S)-Ser(S)-Lys4-OH Trihydrocloride (Pam3Cys) (EMC microcollections, Tübingen, Germany), and extracts from *A. versicolor* (Allergon, Ängelholm, Sweden), and Zymosan A (Sigma Aldrich, Steinheim, Germany) were used as stimuli. *A. versicolor* extracts were prepared by mixing 500 mg substance with 10 mL Aqua *bidest* (50 mg/mL), followed by shaking for 1 h. Afterwards, the samples were autoclaved at 120°C, centrifuged (10 min at 3000g), and the resulting supernatant stored at −80°C. Zymosan A extracts were prepared by suspending 1% by weight in 0.9% NaCl solution, heating for 1 h in a water bath at 37°C, followed by centrifugation for 30 min by 4000 rpm. The pellet was resuspended to 5 mg/mL in 0.9% NaCl solution. Before using the preparations in the whole blood assay (WBA), extracts were heated at 80°C for 30 min.

### Peripheral blood

Heparinized venous fresh blood was collected in the morning (8-10 a.m.) from all volunteers participating in the study in Institute of Prevention and Occupational Medicine (IPA). Fresh blood cells were counted and blood smears were stained by May-Grünwald-Giemsa. Before use in WBA, the blood was stored for 1 h at room temperature. In serum, total IgE, specific IgE to a variety of environmental allergens (sx1 Phadiatop (dog and cat dander; house dust mite, timothy grass, rye, *Cladosporium herbarum*, birch, mugwort), the mould mixture mx1 (*Penicillium chrysogenum*, *Cladosporium herbarum*, *Aspergillus fumigatus, Alternaria alternata*), and specific IgG to the mould mix Gmx6 (*Penicillum chrysogenum*, *Cladosporium herbarum*, *Mucorracemosus*, *Alternaria alternata*) were measured by ImmunoCAP (ThermoFisher Scientific, Uppsala, Sweden) according to the manufacturer’s recommendations. Specific IgE values ≥0.35 kU/L and specific IgG values ≥30 mg/L were considered positive.

### Markers in serum and plasma

Soluble CD14, LBP and BPI were measured in plasma by using ELISA Kits (Hycult biotech, Uden, Netherlands). The detection range for sCD14 was 1.56 - 100 ng/mL, for LBP 4.4 – 50 ng/mL and 102-25000 pg/mL for BPI. In serum IL-6 and IL-8 were quantified by DuoSet ELISA Kits (R&D systems, Wiesbaden, Germany) with the detection range 4.7 - 600 pg/mL and 3.1 - 200 pg/mL, respectively. The detection range for CC16 ELISA Kit (BioVendor, Heidelberg, Germany) was 2 - 100 ng/mL. All ELISAs were performed in accordance to the manufacturer’s instructions. 

### Whole blood assay (WBA)

In the whole blood assay, mediators such as IL-1β, IL-8 or TNF-α released by blood cells in response to incubation with stimulus were measured [7,8]. In the WBA, different final concentrations were used for *E. coli* endotoxin (40; 1000 pg/mL), Zymosan A extract (10; 40 µg/mL), Pam_3_Cys (500 ng/mL) and *A. versicolor* extract (500; 1000 µg/mL). For WBA, 100 µL fresh blood was displaced with 500 µL RPMI following by a pre-incubation step for 1 h at 37°C and 5% CO_2_. After the 1 h incubation step, 300 µL RPMI and 100 µL of the stimulus dilution were added, and the entire mixture incubated for 20 h at 37°C and 5% CO_2_. After centrifugation (2 min, 10000g), cell-free supernatants were aliquoted and frozen at −80°C until further analysis. The released cytokines, IL-1β and IL-8 were measured in the cell-free supernatant by using monoclonal “sandwich” enzyme-linked-immunosorbent (ELISA)-assay kits (IL-1β: DuoSetTM ELISA Development system; R&D Systems, Wiesbaden, Germany; IL-8: Becton Dickinson, Heidelberg, Germany) with a sensitivity range from 3.9 to 250 pg/mL for IL-1β, and 3.1 to 200 pg/mL for IL-8. ELISAs were performed according to the manufacturer’s instructions. All samples were measured in two to three different dilutions and the intra-assay results were accepted if the coefficient of variation (CV) was below 25%. The cytokine release was adjusted for cytokine concentration released in the absence of further stimuli (RPMI medium control). 

### Surface markers on peripheral blood monocytes

The cell surface receptor expression was measured in fresh blood cells one hour after blood drawing as well as after 22 h stimulation of the blood at 37°C and 5% CO_2_ with 40 pg/mL *E. coli e*ndotoxin; 10 µg/mL Zymosan A; 500 pg/mL Pam_3_Cys, 500 µg/mL *A. versicolor* and RPMI as control. For this purpose, blood was incubated with excess of anti-CD14-PerCP, anti-CD45-FITC (BD Bioscience, Heidelberg, Germany) and either anti-TLR-2-PE, anti-TLR-4-PE, anti-dectin-1-PE or isotypematched antibodies as matched negative controls (R&D systems, Wiesbaden, Germany) for 20 min in the dark. Red blood cells were lysed with FACS lysing solution (BD Bioscience, Heidelberg, Germany), followed by washing twice with PBS. All samples were analyzed on a FACSCalibur (BD Bioscience, Heidelberg, Germany). For analysis, CD14- and CD45-positive monocytes were initially gated, followed by measurement of the fluorescence of receptor antibodies or isotype control on these monocytes.

Results are presented as mean relative fluorescence intensity (mRFI) (antibodies - matched isotype control). mRFI values of stimulated samples are additionally adjusted for RPMI control values.

### Statistics

Data were analyzed using GraphPad Prism (GraphPad Software Inc., San Diego, CA, USA). The variables IL-8 and IL-1β were log-transformed in the graphs in order to make the pattern of the data more visible. Results are described with the robust measures median and interquartile range. Statistical analyses were performed by analysis of variance considering atopic and smoking status or gender using SPSS software version 20 (IBM SPSS Statistics, Armonk, NY, USA). In case of values below the detection limit, Tobit regression was applied using SAS/STAT software version 9.3 (SAS Institute Inc., Cary, NC, USA). In order to meet the assumptions of these models and tests, data for non-stimulated TLR-2 and TLR-4 expression and Pam3Cys induced IL-1β release were log-transformed. 

## Results

### Study Group

The mean age of subjects exposed to moisture damage and subjects without exposure was 30 and 35 years, respectively (Table 1). The subjects were predominantly female. The percentage of smokers in the exposed to non-exposed group was 12% and 16%, respectively. Nine subjects out of each group were atopic with sx1 values higher than 0.35 kU/L. No significant difference between both groups was observed. Antigen against *A. versicolor* was detected via specific ELISA in three and *P. chrysogenum* in one room of the 18 rooms with moisture damage that was measured with EDC (Table 2). The median β-(1,3)-glucan concentration in the EDC extracts was about 6.1 µg/m^2^ and the endotoxin activity about 186.6 EU/m^2^.

**Table 2:** Characteristics of electrostatic dust fall collector (EDC) extracts (n=18) of moisture damaged rooms.

	**Results of EDC extracts (n=18) of moisture damaged rooms**
β-(1,3)-Glucan [µg/m^2^]	6.1 (1.6-14.3)
Endotoxin activity [EU/m^2^]	186.6 (134.0 - 502.4)
*A. versicolor*:	
(*A. versicolor* antigen ≥ 38 ng/m^2^ = positive) Positive number: n (%)	3 (17%)
*P. chrysogenum*:	
(*P. chrysogenum* antigen ≥ 77 ng/m^2^ = positive) Positive number: n (%)	1 (6%)

Values for β-(1,3)-Glucan and endotoxin activity in 18 EDC extracts are shown as median and interquartile range.

### Blood Parameters

**Figure 1:**
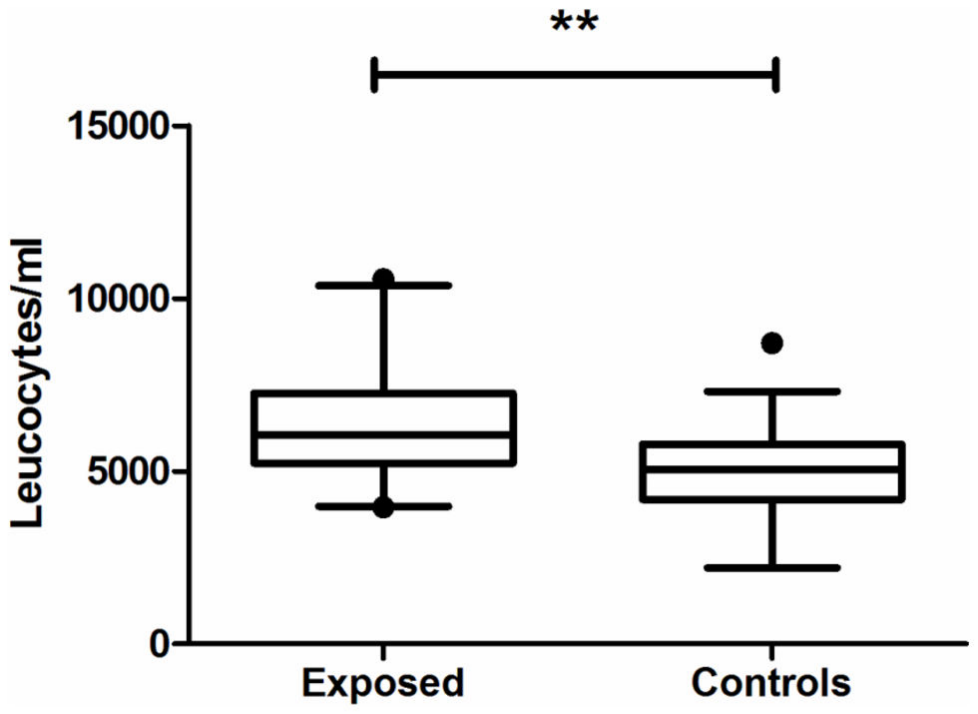
Leucocytes. Number of leucocytes in subjects exposed to moisture damage (n=25) and subjects not exposed to moisture damage (controls; n=25). Statistically significant changes are indicated as **p<0.005 compared with control subjects. The box shows the median and interquartile range, whisker corresponds to 1.5 times the interquartile range.

The number of leucocytes was significantly (p<0.005) higher in exposed compared to control subjects (Figure 1). This difference still remained even after accounting for gender, smoking and atopy status of the subjects. The mean number of leucocytes in exposed subjects was 6350 cells/mL, but only 5100 cells/mL in control subjects. The differential cell counts were without conspicuous features and showed no differences between the groups.

In addition to the cellular distribution, soluble markers in serum (IL-6, IL-8 and CC16) and plasma (BPI, LBP and sCD14) were measured (Table 3). In sera from 22 exposed subjects and 15 control sera, IL-6 concentration was under the detection limit. Furthermore, in 12 plasma samples from exposed and 8 plasma samples from control subjects, the BPI level was not within the detection range, and CC16 quantification was not possible in 3 exposed and 2 control subjects’ sera. Regarding the quantified markers, no significant differences could be observed between both groups.

**Table 3:** Marker in serum and plasma.

**Parameter**	**Exposed**	**Controls**
CC16 [pg/mL]	6.7 (3.7 - 9.0)	7.2 (5.0 - 8.7)
IL-6 [pg/mL]	<4.7 (<4.7 - 7.4)	<4.7 (<4.7 - 23.9)
IL-8 [pg/mL]	21.7 (11.0 - 38.9)	16.5 (11.4 - 34.4)
BPI [pg/mL]	3201 (<408 - 4741)	2310 (<408- 4102)
LBP [µg/mL]	19.9 (15.8 - 25.2)	22.0 (15.8 - 29.2)
sCD14 [µg/mL]	1.3 (1.0 - 1.8)	1.2 (1.1 - 2.1)

IL-6, IL-8, CC16 in serum and LBP, BPI, sCD14 in plasma in subjects exposed to moisture damage (n=25) and subjects not exposed to moisture damage (controls; n=25). Values are shown as median and interquartile range.

### Whole blood assay

**Figure 2:**
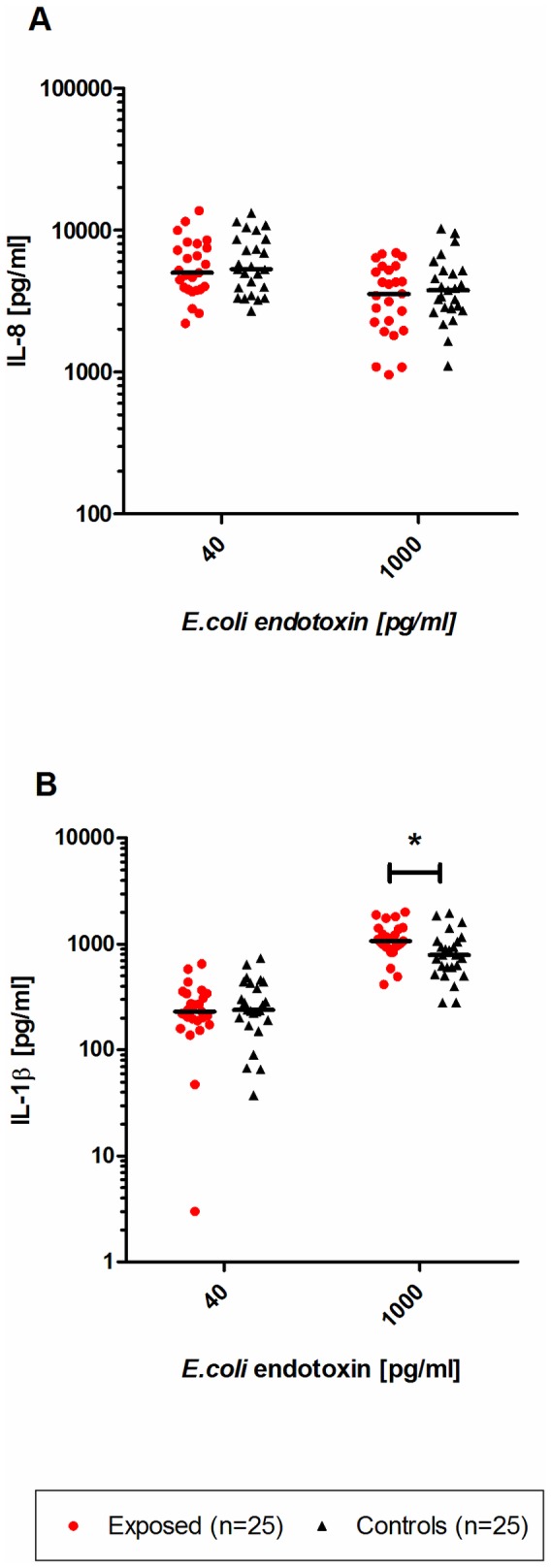
*E. coli* endotoxin induced mediator release. IL-8 (A) and IL-1β (B) response in fresh whole blood after 20 h stimulation with *E. coli* endotoxin in subjects exposed to moisture damage (n=25) and subjects not exposed to moisture damage (controls; n=25). Statistically significant changes are indicated as *p<0.05 compared with control subjects. Bold lines show median values.

The spontaneous mediator release of IL-1β and IL-8 after 18 h incubation with RPMI showed individual variations, but no significant differences between both groups (Table 4). After stimulation with *E. coli* endotoxin (1000 pg/mL), the IL-1β release was significantly increased (p<0.05) in exposed subjects compared to control subjects (Figure 2). The median IL-1β response in control subjects was about 560 pg/mL, whereas the response in exposed subjects was about 930 pg/mL. No differences were found in the release of IL-1β between the groups after fresh blood stimulation with 40 pg/mL *E. coli* endotoxin or concerning the release of IL-8 after endotoxin stimulation. 

**Table 4:** Mediator release after 20 h whole blood stimulation.

**Stimuli**	**Exposed**			**Controls**		
	IL-1β [pg/mL]	IL-8 [pg/mL]	TNF-α [pg/mL]	IL-1β [pg/mL]	IL-8 [pg/mL]	TNF-α [pg/mL]
**RPMI (medium control)**	<3.9 (<3.9 - 4.8)	141.3 (83.1 - 343.3)	<15.6 (<15.6 - <15.6)	<3.9 (<3.9 - 10.0)	97.3 (28.6 - 154.4)	<15.6 (<15.6 - 24.4)
**Pam_3_Cys (500 ng/mL)**	37.8* (16.4 - 110.0)	2552 (916 - 4118)	53.3 (29.9 - 94.5)	7.9 (<3.9 - 32.4)	2593 (1148 - 3983)	26.9 (13.6 - 76.1)
**Zymosan A (10 µg/mL)**	2510 (2104 - 2833)	35853 (21432 - 42960)	1507 (1043 - 1896)	1987 (1677 - 2484)	29097 (16950 - 39958)	1213 (951.1 - 1521)
**Zymosan A (40 µg/mL)**	3993 (3007 - 5240)	28185 (2276 - 35422)	1996 (1490 - 2662)	3753 (2855 - 4023)	24237 (18344 - 37842)	1768 (1325 - 2407)
***E. coli* endotoxin (40 pg/mL)**	230.6 (181.3 - 341.4)	3561 (2105 - 5399)	194.3 (141.2 - 305.6)	238.7 (181.1 - 435.7)	3770 (2757 - 5190)	247.3 (207.2 - 357.6)
***E. coli* endotoxin (1000 pg/mL**)	1067* (932.8 - 1405)	5025 (3817 - 7757)	814.8 (555.0 - 978.2)	787.3 (560.4 - 1066)	5321 (3695 - 8631)	637.3 (493.0 - 780.2)
***A. versicolor* (500 µg/mL)**	<3.9* (<3.9 - 8.8)	3885* (1687 - 8254)	<15.6 (<15.6 - <15.6)	<3.9 (<3.9 - <3.9)	1487 (950 - 2998)	<15.6 (<15.6 - <15.6)
***A. versicolor* (1000 µg/mL)**	5.1* (<3.9 - 7.6)	9927* (5317 - 15097)	<15.6 (<15.6 - <15.6)	<3.9 (<3.9 - <3.9)	4164 (2623 - 6364)	<15.6 (<15.6 - <15.6)

Release of IL-1β and IL-8 in subjects exposed to moisture damage (n=25) and subjects not exposed to moisture damage (controls; n=25) after incubation of whole blood with RPMI, Pam_3_Cys (500 ng/mL), Zymosan A (10 µg/mL, 40 µg/mL), *E. coli* endotoxin (40 pg/mL, 1000 pg/mL) and *A. versicolor* (500 µg/mL, 1000 µg/mL). Values are shown as median and interquartile range. Statistically significant changes are indicated as *p<0.05 compared with control subjects.

The stimulation with Pam_3_Cys also revealed a significant (p<0.05) higher IL-1β release in exposed subjects. The median IL-1β release in exposed subjects was about 37.8 pg/mL, the release in control subjects about 7.8 pg/mL IL-1β. No differences were found in the release of IL-8 between control and exposed subjects. 

**Figure 3:**
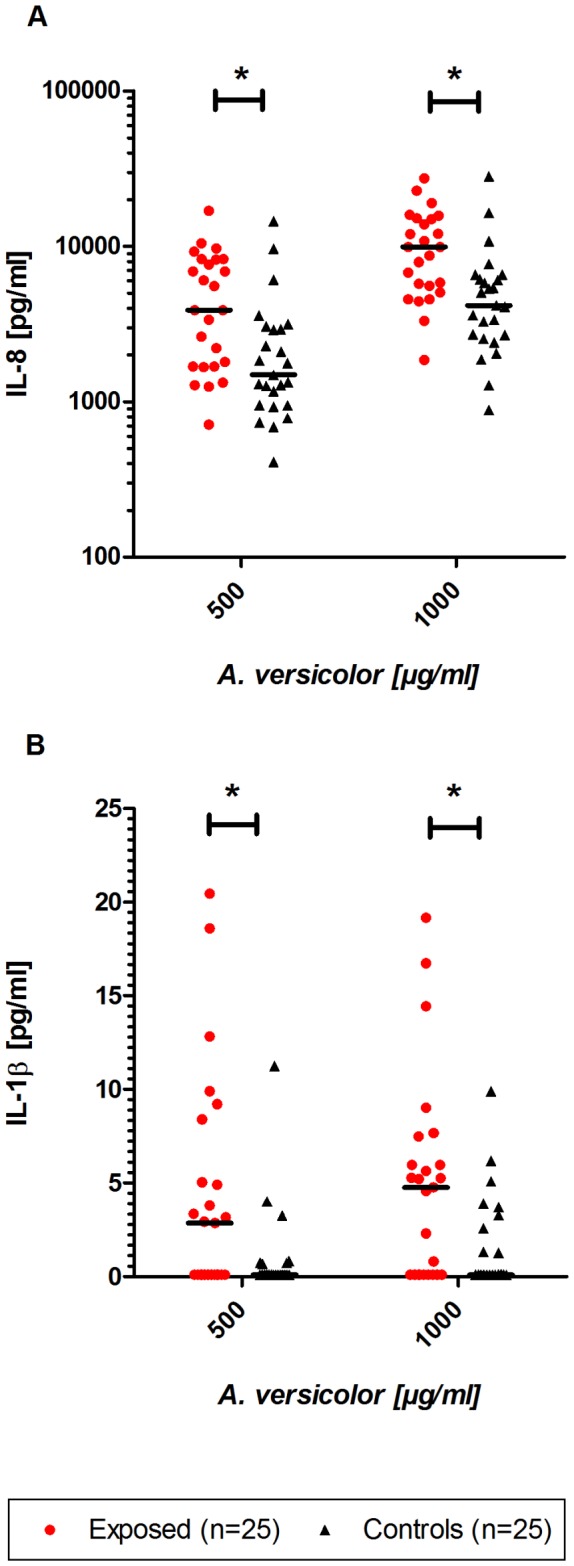
*A. versicolor* induced mediator release. IL-8 (A) and IL-1β (B) response after 20 h stimulation in fresh whole blood with *A. versicolor* in subjects exposed to moisture damage (n=25) and subjects not exposed to moisture damage (controls; n=25). Statistically significant changes are indicated as *p<0.05 compared with control subjects. Bold lines show median values.

In contrast, the IL-8 response after stimulation with both *A. versicolor* concentrations (1000 µg/mL and 500 µg/mL) in fresh blood was significantly higher (p<0.05) in exposed than in control subjects (Figure 3). The median IL-8 release was approximately 5320 pg/mL and 1690 pg/mL, respectively in exposed subjects and 2620 pg/mL and 950pg/mL, respectively in control subjects. Although the IL-1β release after stimulation of fresh blood with *A. versicolor* was relatively low, a significantly enhanced response (p<0.005) could be detected in exposed subjects after stimulation with both *A. versicolor* concentrations. In exposed subjects, the median IL-1β release was about 5 pg/mL and 3 pg/mL, respectively after stimulation with *A. versicolor* (1000 µg/mL and 500 µg/mL). The release after stimulation with *A. versicolor* in control subjects was under the detection limit.

The stimulation with Zymosan A revealed no differences in release of IL-1β or IL-8 (Table 4). Also, the quantification of TNF-α response induced by stimulation with the various stimuli showed a similar release in both groups (data not shown). All differences in the WBA experiments were independent of gender, smoking and atopy status tested via multiple regression analysis.

### TLR-2, TLR-4 and dectin-1 expression on monocytes

**Figure 4:**
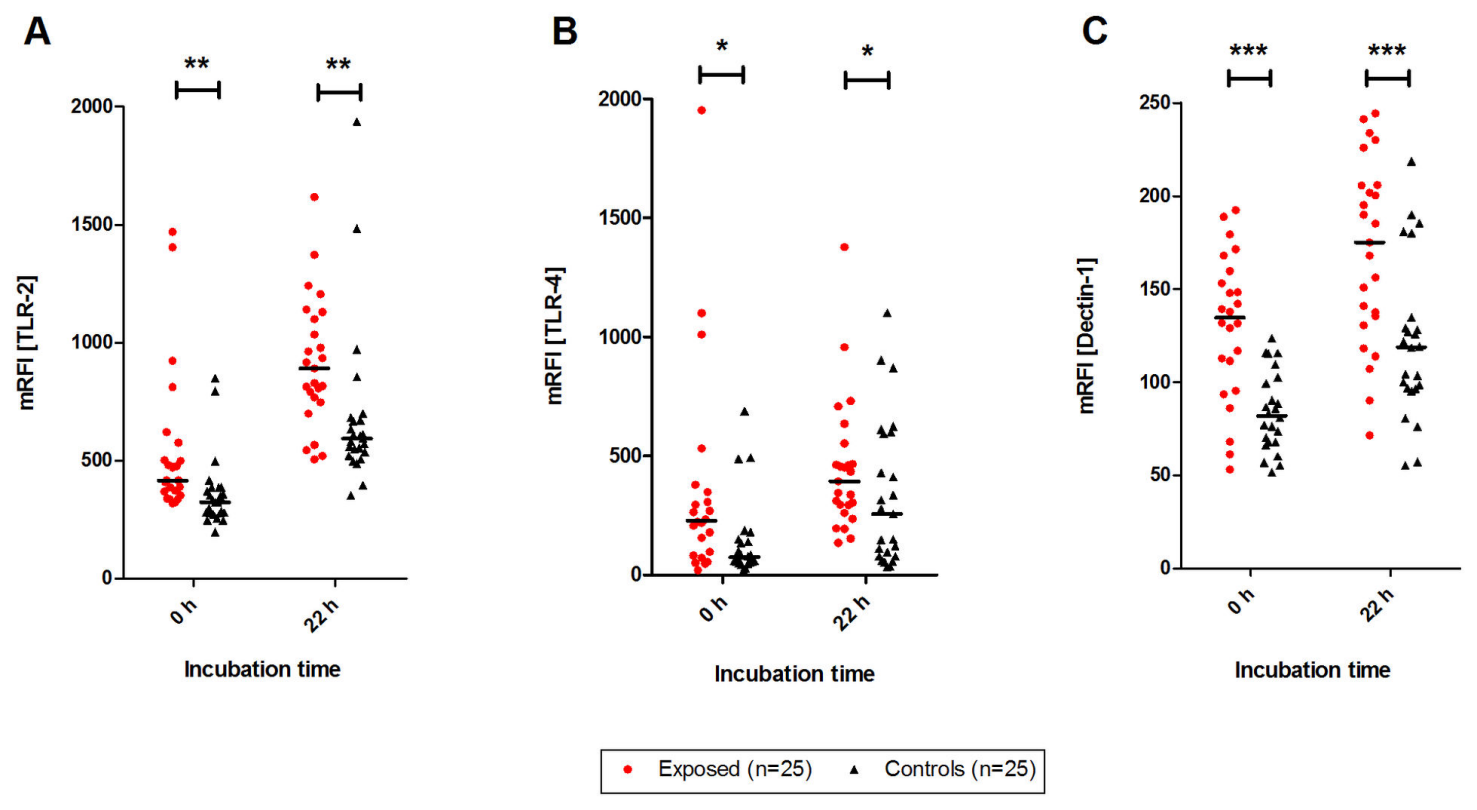
TLR-2, TLR-4 and dectin-1 expression. Expression of TLR-2 (A), TLR-4 (B) and dectin-1 (C) on monocytes of fresh and 22 h RPMI-incubated whole blood in subjects exposed to moisture damage (n=25) and subjects not exposed to moisture damage (controls; n=25). Mean relative fluorecence intensity (mRFI) is demonstrated. Statistically significant changes are indicated as *p<0.05, **p<0.005 and ***p<0.0005 compared with control subjects. Bold lines show median values.

In fresh blood, the TLR-2 (p<0.005), TLR-4 (p<0.05) and dectin-1 (p<0.0005) cell surface expression on monocytes of exposed subjects was significantly enhanced compared to the expression on monocytes of control subjects (Figure 4). In addition, after 22 h incubation with RPMI medium, the TLR-2 (p<0.005), the TLR-4 (p<0.05) and the dectin-1 (p<0.0005) expression were still significantly higher in exposed than in control subjects. 

Stimulation with *E. coli* endotoxin, Zymosan A and Pam_3_Cys for 22 h caused increased TLR-2, TLR-4 and dectin-1 expressions, whereas the stimulation with *A*. versicolor only increased the expression of TLR-2 and TLR-4. In contrast, the dectin-1 expression induced by *A. versicolor* was decreased (Table 5). All these effects were observed in both groups and were independent of the exposure to moisture damage by the subjects in their dwellings. 

**Table 5:** Expression of TLR-2, TLR-4 and dectin-1 on monocytes after 22 h stimulation.

	**TLR-2**		**TLR-4**		**dectin-1**	
	Exposed	Controls	Exposed	Controls	Exposed	Controls
**Endotoxin (40 pg/mL)**	329 (226 - 667)	467 (252 - 876)	18 (-44 - 107)	23 (-58 - 216)	31 (6 - 77)	25 (6 - 67)
**Zymosan A (10 µg/mL)**	955 (637 - 1144)	845 (645 - 1281)	54 (-81 - 149)	122 (35 - 403)	53 (-12 - 113)	60 (21 - 134)
**Pam_3_Cys (500 ng/mL)**	510 (188 - 820)	409 (90 - 967)	27 (-71 - 339)	25 (-68 - 485)	30 (4 - 103)	6 (-13 - 53)
***A. versicolor* (500 µg/mL)**	624 (239 - 1333)	313 (228 -636)	110 (-39 - 205)	22 (-54 - 90)	-53 (-95 - -19)	-38 (-54 - -8)

Expression of TLR-2, TLR-4 and dectin-1 after stimulation with *E.coli e*ndotoxin, Zymosan A, Pam_3_Cys and *A.versicolor* in whole blood of subjects exposed to moisture damage (n=25) and subjects not exposed to moisture damage (controls; n=25). All values are mean relative fluorescence intensities (mRFI). The values are emanated from mRFI of medium control. Data are shown as median and interquartile range.

## Discussion

Adverse health effects of dampness and moulds in homes have been reported [4,28], but previous studies have failed to fully evaluate the effect of such exposures on the innate immunity. Our study focused on *ex vivo* effects assessed by detailed analyses of cellular marker and cytokine releasability comparing subjects with and without moisture damage exposure. Our results demonstrated an enhanced number of leucocytes in the blood of subjects exposed to moisture damage compared to control subjects, indicating a slightly increased reactivity of the immune system. However, the number of leucocytes was within the normal range, showing there is no acute inflammation in the subjects. In addition, the percentage of the leucocytes estimated via differential blood staining was within normal range and without differences between both groups. The concentrations of further soluble blood markers such as CC16, a marker of respiratory diseases and the inflammatory markers IL-6 and IL-8 were also similar in both groups. Zhang et al. describes that exposure to dampness or moulds at the workplaces at baseline was negatively associated with IL-6 release in the 10 years-follow up investigations [29]. However, in accordance with our results, they found no association between continued exposure and IL-6 level. Our investigations also showed no differences between control and exposed subjects regarding the level of the endotoxin recognition involved proteins LBP, BPI and sCD14. Borm et al. demonstrated an increased level of BPI in 20 animal keepers, who were exposed to high levels of organic dust, compared to 20 matched non-exposed controls. Similar to our results, the LBP concentration was not different between both groups [30]. Also, investigations with 15 pig farmers, who were exposed to high endotoxin levels, showed no difference in sCD14 levels compared to 15 non-exposed controls [31]. Admittedly, the bioaerosol exposure of animal keepers and farmers is quite different compared to that obtained in rooms with moisture damage in terms of qualitative and quantitative composition. Endotoxin and β-(1,3)-glucan concentrations in EDC extracts of rooms with moisture damage were comparable to concentrations measured by Noss et al. in homes without moisture damage on EDC filters extracted without Tween [32]. 

The WBA exhibited an increased IL-1β release in exposed subjects after stimulation with 1000 pg/mL *E. coli* endotoxin and 500 ng/mL Pam_3_Cys, whereas the IL-8 and TNF-α release were not affected. Possibly, the increased response to endotoxin and Pam_3_Cys as shown in our results can be attributed to increased exposure to moisture damage. Smit et al. indicated an association between airway symptoms and an increased IL-10 and TNF-α response in 412 agricultural workers after *ex vivo* stimulation with *E. coli* endotoxin in whole blood. In addition, subjects with a high cytokine release within the collective showed a significant association between individual endotoxin exposure in stable and IL-1β and IL-10 responses [11]. Sahlander et al. demonstrated no differences in IL-6 and TNF-α release in 15 farmers compared with 15 controls after stimulation of whole blood with *E. coli* endotoxin, Pam_3_Cys or stable dust, although farmers had an increased number of monocytes [31]. In contrast to the results of our study, Beijer et al. demonstrated no differences in endotoxin-induced IL-1β-, TNF-α-, IFN-γ-, IL-4- and IL-10-release in high β-glucan-exposed subjects compared to lower-exposed subjects [33]. The β-glucan level in rooms was used as a marker of mould exposure. However, it should be considered that Beijer et al. used isolated peripheral blood monocytes (PBMCs), whereas our experiments were done with whole blood. . In addition, the β-glucan level may not reflect mould exposure optimally.

In our study, a significant increase in IL-1β and IL-8 release from whole blood cells in exposed subjects induced by *A. versicolor* could be detected. *A. versicolor* is one of the most common moulds found indoors after moisture damage [2,22]. For this reason, inhabitants of moisture damaged rooms are most likely exposed to components of *A. versicolor*. Our results suggest that the indoor exposure to *A. versicolor* affects the mediator release of cells. In other studies, the mediator release was higher in exposed subjects when the stimulation occurred together with the exposure substance, for example in animal keepers or fish factory workers [30,34]. In our study, *A. versicolor* antigen could be detected in three dust extracts out of 18 collected in rooms of subjects exposed to moisture damage. Analysis of surface samples by other studies have already shown an occurrence of *A. versicolor* in 72% of all households with moisture damage [2]. The low proportion of *A. versicolor* verification in our study may have two reasons. Firstly, no formation on *A. versicolor* was present in the rooms with moisture damage, and secondly, our exposure assessment strategy using EDC samplers was not sufficient for these exposure circumstances because only airborne antigens are detectable. Other factors such as aerosolized substances could also play an important role in the formation of immunological effects in exposed subjects. Immunmodulatory effects have already been confirmed for the cell wall component β-glucan and the toxic metabolite of *A. versicolor*, Sterigmatocystin [35,36]. 

The expression of TLR-4 as well as TLR-2 and dectin-1 on the cell surface of monocytes in fresh blood in our study was increased in exposed subjects compared to control subjects. Even after 22 h of storage with RPMI, both TLR-2 and dectin-1 expressions were still increased in the exposed subjects. The stimulation of whole blood with the model components *E. coli* endotoxin, Zymosan A, *A. versicolor* and Pam_3_Cys caused no significant differences in TLR-2, TLR-4 and dectin-1 expression on monocytes from exposed subjects compared to the controls.

One reason for the increased cell surface expressions of TLR-2, TLR-4 and dectin-1 on monocytes of exposed subjects could be the daily exposure to possible receptor ligands in the rooms with moisture damage. The typical TLR-2 ligands include lipoproteins from Gram-positive bacteria such as actinomycetes, which can often be detected in bioaerosols of moisture damaged rooms [16]. Endotoxin and β-1,3-glucan, as typical TLR-4 and dectin-1 ligands were also shown to be increased in dwellings with moisture damage [3]. Furthermore, the expression of the innate immune system in cells is regulated by inflammatory mediators [37]. 

Sahlander et al. showed a decreased TLR-2 expression on fresh blood monocytes of eleven non-smoking bioaerosol-exposed farmers compared with 12 non-smoking control subjects. However, no differences in TLR-4 expression were detected. Neither bronchial inhalation of endotoxin nor 3 h of farm work in a stable resulted in differences in TLR-2 and TLR-4 expression on monocytes between farmers and controls [38]. Our results showed increased TLR-2, TLR-4 and dectin-1 expression after stimulation with *E. coli* endotoxin, Zymosan A and Pam_3_Cys, whereas the stimulation with *A*. versicolor only increased the expressions of TLR-2 and TLR-4. In contrast, *A*. *versicolor* decreased dectin-1 expression.

Many other studies have already examined the effects of stimulation with endotoxin, Zymosan A or *A. fumigatus* on TLR-2, TLR-4 and dectin-1 expression [39–41]. However, these studies provide conflicting results with regard to the influence of the stimulants on receptor expression. Possible reasons for the divergent results of these studies can be attributed to the different stimulation durations, and concentrations of the stimuli used. In studies on the kinetics of TLR-4 expression, a bell-shaped curve of TLR-4 expression was detected over a 24 h period stimulation with endotoxin [42]. Furthermore, various cellular systems such as human PBMCs or bronchial epithelial cells were used, and so the results cannot be completely compared to our results. Moreover, if receptors are internalized during activation, measurement is not possible, and therefore, both an increase and a decrease in receptor expression may be a sign of activation.

Overall, we give a hint that exposure to bioaerosols from rooms with moisture damage alters the *in vitro* stimulated mediator releaseability and the expression of the receptors TLR-2, TLR-4 and dectin-1. Despite the small number of subjects, 25 exposed and 25 non-exposed subjects to moisture damage, this study is an initial proof of concept because it is the first evidence available that exposure to moisture damage can alter characteristics of the innate immune system.
